# New Drugs, Appliances, and Things Medical

**Published:** 1892-03-19

**Authors:** 


### NEW DRUGS, APPLIANCES, AND THINGS MEDICAL.

[All preparations, appliances, novelties, etc., of which a notice is desired, should be sent for The Editor, to care of The Manager, 140, Strand, London, W.C.]

### LANURA FLANNEL.

Flannel is becoming increasingly used as underclothing, as the wisdom of its adoption is becoming indisputable. Manufacturers have realised that the grave objection to it was its tendency to cause irritability of the skin, and have, therefore, confronted the difficulty with success. Mr. Williamson, of 91, Edgware Road, is especially happy in offering the public all that it is possible in the way of comfort, durability, and cheapness in his Lanura Flannel. An excellent make is to be had for the low price of 11½d. the yard, whilst for 1s. 6d. we have a material as soft as silk, and of evident durability. A special manufacture, 80 inches wide, of wonderful evenness and closeness of texture, and most suitable for dressing-gowns and other articles of clothing where the narrower materials cut to waste. Some neat and pretty fancy "Lanuras" are supplied at 1s. 4½d. the yard. Mr. Williamson is very obliging in sending patterns, for which we advise our readers to apply.

### BRAIDED WIRE CUSHIONS AND SEATS.

The American Braided Wire Company have supplied us with samples of their novelties in cushions, which we are glad to have had an opportunity of inspecting. Wire is not suggestive of comfort, nevertheless we can unhesitatingly say that we have not come across anything so luxurious and supporting at the same time as the best of these articles. To rest against it is soft as a down cushion, but is in other respects infinitely superior owing to the elasticity of the manufacture, and on the score of hygiene by the possibility of air being passed through the cushion, it is unrivalled. The less fine makes of the same manufacture are only slightly less comfortable, and their adaptability to all sorts of purposes is quite surprising. One neat little travelling cushion, supplied with serviceable covering and strap handle, is really admirable, and so light as to be a hardly perceptible burden. The material is especially adapted for opera chairs, and any theatre adopting these will greatly add to the comfort of an audience. In fact, in any circumstances where prolonged sitting is an attendant feature, fatigue will be minimised. It is difficult to speak too highly of the products of the American Braided Wire Company, and a visit to their depôt at 64, Church Street, Shoreditch, will show that we have not praised unduly.

### SACKLIN'S PATENT POULTICES.

As the practice of medicine is still conducted in this country it is impossible to do without a poultice, although this form of external medication is very far from an ideal one. Everybody who has made a poultice knows that it is a troublesome, dirty, and sometimes a tedious process, necessitating basins, packets of linseed meal, a knife to stir with, old linen to spread the mass on, boiling water, and a dish to facilitate the smoothing out of the mass. Although there is nothing after all so comforting as a well made poultice, yet it is at best an archaic application. Messrs. Sacklin have patented a great improvement in this method of treatment. A definite quantity of linseed meal is placed between a layer of muslin and one of waterproof calico. The edges are sewn together and the area of poultice material divided off by seams into regular sized squares. Thus, if the original poultice is too big, by cutting off a piece between the seam the desired size can be obtained. All that is required is to pour a sufficiency of boiling water on them from the kettle and after drying up superfluous moisture on a towel to apply the poultice to the skin. If the skin be unbroken, the poultice can be used over and over again to the extent of from twenty to thirty times. The same method of manufacture is used in the making of the patent mustard poultices. Real mustard is employed and the peculiarly disagreeable pungency of mustard papers is obviated, owing to the presence of the ground mustard husks, which, containing a larger proportion of bland oil, tend to prevent blistering, while, at the same time, the desired reaction is not interfered with. We consider that the invention is a distinct advance in the application of the therapeutics of external counter-irritation.

### BLANKETS.

Messrs. Hardman and Sons, of Bury, Lancashire, make such a speciality of blankets, that we call the attention of those who have to cater for institutions, to their manufactures. These blankets are supplied of every possible quality and at wonderfully moderate prices, the profits of the middleman being dispensed with. Messrs. Hardman are willing to enter into contract for large supplies, and execute any design required. They will even manufacture customers' own wool, whilst their own specialities range from the gaudy articles likely to prove so useful to missionaries or others desirous of pleasing native taste, to the very finest textures fit for use in royal households. The "Charity" blanket of warm and cheerful appearance is offered for the small sum of three shillings, whilst the "Stanley" blanket should be welcome in the nursery for its qualities of lightness, softness, and warmth. Although the Fernhill Mills are noted for their blankets, we were much struck with the patterns of other woollen materials, especially of fine cloths, which are of undoubted excellence.

